# In Memoriam

**Published:** 1888-01

**Authors:** 


### In Memoriam.

Dr. A. B. Palmer, dean of the medical department, and professor of pathology and practice of medicine in the University, died at his home in Ann Arbor, Dec. 23, at 8 P. M.

BIOGRAPHY.

Prof. Alonzo Benjamin Palmer, A.M., M.D., L.L.D., was born in Richfield, N. Y., Oct. 6, 1815. His ancestors were among the earlier English Puritans and Hollanders who settled in New England and Eastern New York. His father died when Dr. Palmer was only nine years old. Dr. Palmer obtained his education in the common schools and in the select schools of Otsego, Herkimer and Orange counties in New York. He studied medicine in the New York College of Physicians and Surgeons, where he graduated in 1839. Afterwards he studied in Philadelphia and in Europe. In 1850 he removed from Tecumseh, Mich., to Chicago. Here he was city physician in 1852 during a severe epidemic of Asiatic cholera among immigrants from Norway, Sweden, and Germany. The cholera hospital was under his charge, and he made a valuable report on the subject of cholera. About 1,500 cases of cholera came under his notice.

Prof. Palmer in 1852 was offered the chair of anatomy in the U. of M., but did not come until 1854. Dr. C. L. Ford and he entered the University as lecturers at the same time, in October, 1854. Dr. Palmer was next made professor of materia medica and therapeutics, and in 1869 he was given the chair which he held to the time of his death, that of pathology and practice of medicine. He was dean of the department of medicine and surgery.

Upon the opening of the Rebellion, Prof. Palmer went to the front as surgeon of the 2nd Michigan Infantry, and was in Gen. Richardson's brigade at the first battle of Bull Run. He dressed the first wound inflicted by the enemy at Blackburn Ford on July 18, 1861. In September he returned to his lecturing, spending his vacations with our armies at the front. In 1864 he was appointed professor of pathology and practice of medicine in the Berkshire Medical College in Pittsfield, Mass., and in 1869 he was called to a similar place in the medical school at Bowdoin College, Maine. These lectures were given during his vacations here. Many thousand persons have listened to his lectures. It was while in Pittsfield that he was married in 1867, to the lady who now mourns his loss.

Prof. Palmer's principal work is a Treatise on the Science and Practice of Medicine, in two volumes, published in 1883. It is one of the most complete works in the English language. He was chief editor for seven years of the *Peninsular Journal of Medicine.* He was a somewhat voluminous writer of books and pamphlets, the last of which, his book on cholera, was printed in THE REGISTER office. In 1860 he was made Vice-President of the American Medical Association, and was at one time a delegate to the British Medical Association. He was President of the Section on Pathology, of the Ninth International Medical Congress, held at Washington, D. C., September 5th, 1887. Through his energetic efforts, this section was one of the most successful and interesting of the Congress.

